# ﻿A new species of *Lecanodiaspis* Targioni Tozzetti, 1869 (Hemiptera, Coccoidea, Lecanodiaspididae), with an updated checklist of the false pit scales of China

**DOI:** 10.3897/zookeys.1240.148728

**Published:** 2025-06-06

**Authors:** Jiangtao Zhang, Keqing Wang, Gillian W. Watson, Xingping Liu, Xubo Wang

**Affiliations:** 1 Jiangxi Provincial Key Laboratory of Conservation Biology, College of Forestry, Jiangxi Agricultural University, Nanchang 330045, China; 2 College of Forestry, Jiangxi Agricultural University, Nanchang 330045, China; 3 Key Laboratory of Forest Disaster Warning and Control in Yunnan Province, College of Forestry, Southwest Forestry University, Kunming 650224, China; 4 130th Regiment of the 7th Division of Xinjiang Production and Construction Corps, Huyanghe 834034, China; 5 Science: Research, Natural History Museum, Cromwell Road, London SW7 5BD, UK; 6 Yunnan Academy of Biodiversity, Southwest Forestry University, Kunming 650224, China

**Keywords:** Distribution, Fagaceae, host, key, *Lecanodiaspisjiangxiensis*

## Abstract

A new species of false pit scale (Hemiptera, Coccoidea, Lecanodiaspididae), *Lecanodiaspisjiangxiensis* Zhang & Wang, **sp. nov.**, collected on *Castaneamollissima* Blume (Fagaceae) in Jiangxi, south China, is described and illustrated based on the morphology of the adult female. A taxonomic key and an updated checklist of false pit scales known from China are provided.

## ﻿Introduction

The scale insects (Hemiptera: Sternorrhyncha: Coccoidea) are small, sap-sucking true bugs (Hodgson and Hardy 2013), with more than 8500 described species across 35 extant and 20 extinct families (García Morales et al. 2016). The family Lecanodiaspididae (Hemiptera: Coccoidea) was established by Borchsenius (1959), with *Lecanodiaspis* Targioni Tozzetti as the type genus. This genus was initially placed in the family Asterolecaniidae but was elevated to family rank by Borchsenius (1959), primarily due to the presence of an anal cleft. Currently, Lecanodiaspididae is composed of 83 species in 12 genera (García Morales et al. 2016), although the validity of some genera remains in doubt (Wang et al. 2024).

The Lecanodiaspididae, also known as false pit scales, are widespread across all zoogeographical regions, with the highest diversity in the Oriental and Australasian regions (Wang et al. 2024). According to ScaleNet (García Morales et al. 2016), an open-source database for scale insects, China has 16 species of false pit scales in five genera: *Cosmococcus* Borchsenius, 1959 (one species); *Crescoccus* Wang, 1982 (one species, considered a synonym of *Pseudopulvinaria* Atkinson, 1889 (family Coccidae) by Tang and Hao 1995); *Lecanodiaspis* Targioni Tozzetti, 1869 (six species); *Prosopophora* Douglas, 1892 (five species, considered a synonym of *Lecanodiaspis* by Cockerell 1896, Howell and Kosztarab 1972); and *Psoraleococcus* Borchsenius, 1959 (three species). However, through the examination of specimens and a review of the literature, four additional species have been recorded or found in China: (i) *Anomalococcuscrematogastri* Green, 1902, reported from Taiwan by Lambdin and Kosztarab (1973); (ii) *Psoraleococcuslombokanus* Lambdin & Kosztarab, 1973, reported from Fujian and Sichuan by Tang and Hao (1995); (iii) *Lecanodiaspisquercus* Cockerell, 1896, was collected from Yunnan; and (iv) *Psoraleococcusmultipori* (Morrison, 1921) was collected from Hainan. On the other hand, *Crescoccuscandidus* Wang, 1982 was not included in this study, and *Lecanodiaspismajestica* Wang & Qiu, 1986 was treated as a junior synonym of *L.quercus*; detailed explanations for both these cases are provided in the Discussion below. This brings the total number of false pit scale species recorded in China to 18.

In the present study, a new species of *Lecanodiaspis*, *L.jiangxiensis* Zhang & Wang, sp. nov., collected on *Castaneamollissima* Blume (Fagaceae) in Jiangxi, south China, is described and illustrated based on adult female morphology, increasing the total number of recorded species to 19. We provide an updated checklist of false pit scales known from China, with information on their hosts and distributions and a taxonomic key to the species.

## ﻿Materials and methods

The specimens examined in this study are deposited in the College of Forestry, Jiangxi Agricultural University, Nanchang, China (**CFJAU**), and the Insect Collection of the Southwest Forestry University, Yunnan, China (**SWFU**). In the ‘Type material’ section, holotype data is provided with “/” used to separate each line of information on the slide label, which is written in Chinese.

Freshly collected specimens were carefully placed in tubes containing 75% or 100% ethyl alcohol and transported to the laboratory. The cuticles of the specimens were stained and mounted on microscope slides using the method described by Borchsenius (1950). The morphological terminology used follows Howell (1971) and Lambdin and Kosztarab (1973). The taxonomic illustration of *L.jiangxiensis* includes a central enlargement of the entire body, with the dorsum shown on the left side and the venter on the right, along with vignettes of important structures (not drawn to scale) around the margin. The body measurements are provided in millimeters (mm), while those for other structures are given in microns (µm). In the checklist, genera and species are listed alphabetically, accompanied by relevant references, host plant records, and collecting sites in China.

## ﻿Results

### ﻿Taxonomy

#### ﻿Genus Lecanodiaspis

Taxon classificationAnimaliaHemipteraLecanodiaspididae

Targioni Tozzetti, 1869

EAF3F9E0-FAFE-528E-A454-EBF8487B480D

##### Type species.

*Lecanodiaspissardoa* Targioni Tozzetti, 1869 by monotypy.

##### Generic diagnosis.

(adapted and modified from Hodgson 1973; Kosztarab and Kozár 1988; Williams and Watson 1990). Adult female test waxy or papery, enclosing adult and eggs. Adult female subcircular or elliptical. Antennae each normally 7–9-segmented. Legs absent or each 1–5-segmented, without tibio-tarsal sclerosis. Thoracic spiracles each associated with primarily quinquelocular pores that extend into spiracular furrow, terminating at enlarged spiracular setae on body margin; usually with two spiracular setae opposite each anterior spiracle; also with a single spiracular seta present opposite each posterior spiracle, except when posterior spiracular furrow divided, then end of each furrow with a single spiracular seta. Dorsum with cribriform plates normally present in two (rarely four) longitudinal rows. Anal ring bearing 8–10 setae and two or three rows of cone- or nipple-shaped pores; with an arched plate above anal ring. Anal plates each usually triangular, ridged and with two stout setae dorsally, plates normally connected at mid-line. Anal cleft short; anal lobes each with single long apical seta and associated short setae. Dorsum with various disc pores, 8-shaped pores, tubular ducts and setae present. Ventral margin with a line of large 8-shaped pores similar to dorsal pores, a submarginal line of smaller 8-shaped pores, and a sparse band of minute bilocular pores reaching as far medially as an imaginary line formed by antennal bases, coxae and anal plates medially to the 8-shaped pores. Minute simple pores and tubular ducts present throughout ventral surface; multilocular disc pores present around genital opening and more anteriorly; quinquelocular pores present in spiracular furrows, sometimes reduced to small groups near spiracles. Labium 1-segmented, with short terminal setae.

#### ﻿ Lecanodiaspis jiangxiensis

Taxon classificationAnimaliaHemipteraLecanodiaspididae

Zhang & Wang sp. nov.

7A1CDEB6-94A5-5A60-8824-D427F1173969

https://zoobank.org/3ECD91AD-EBAB-402A-98D2-AE3FE3D8A1CD

**Figure 1.**
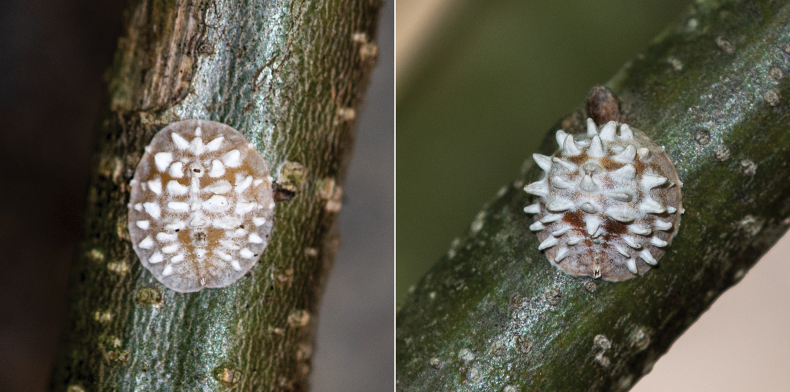
Adult females of *Lecanodiaspisjiangxiensis* Zhang & Wang, sp. nov. on branches of *Castaneamollissima* (Fagaceae).

**Figure 2.**
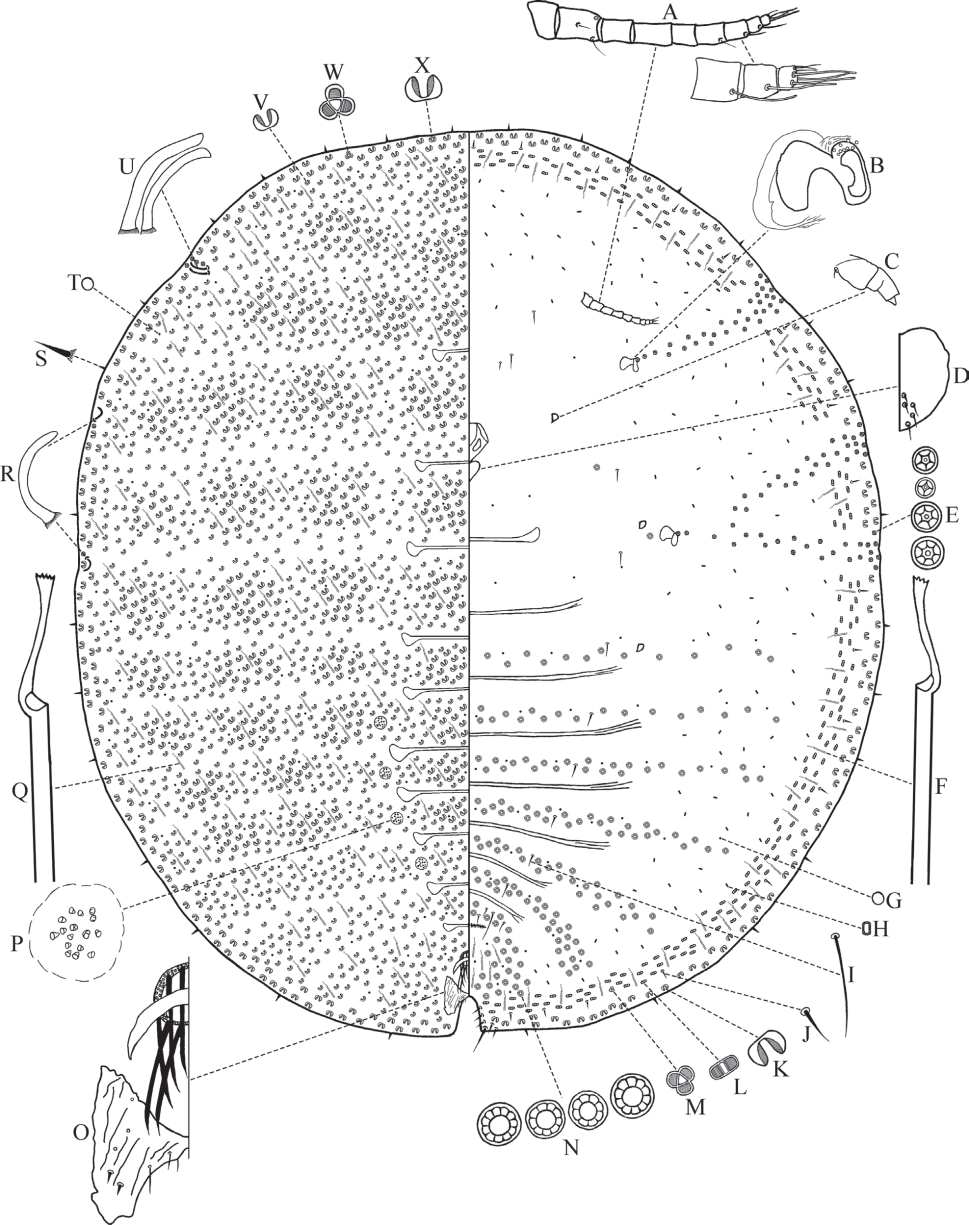
Adult female of *Lecanodiaspisjiangxiensis* Zhang & Wang, sp. nov. **A** antennae **B** spiracle **C** leg **D** labium **E** 5-, 4-, 6-, 7-locular pores **F** ventral tubular duct **G** ventral simple disc pore **H** bilocular pore **I** ventral submedian seta **J** ventral submarginal seta **K** ventral 8-shaped pore **L** ventral flat 8-shaped pore **M** ventral trilocular pore **N** 10-, 8-, 9-, 11-locular pores **O** anal ring, anal plates, arched plate **P** cribriform plate **Q** dorsal tubular duct **R** posterior spiracular seta **S** marginal seta **T** dorsal simple disc pore **U** anterior spiracular setae **V** dorsal small 8-shaped pore **W** dorsal trilocular pore **X** dorsal large 8-shaped pore.

**Figure 3.**
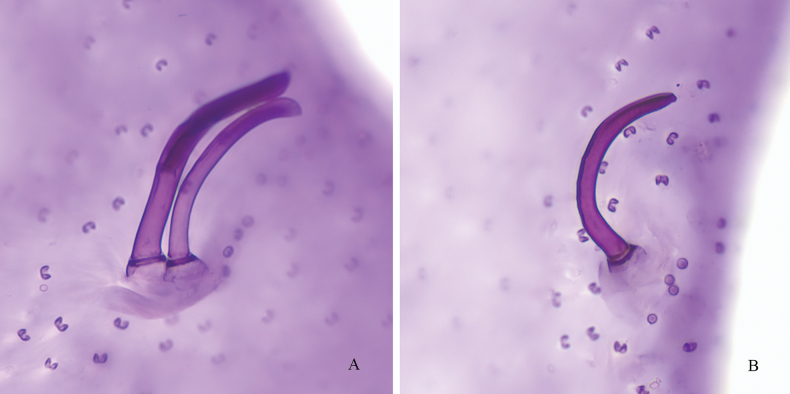
Spiracular setae of *Lecanodiaspisjiangxiensis* Zhang & Wang, sp. nov. **A** two anterior spiracular setae **B** a posterior spiracular seta.

**Figure 4.**
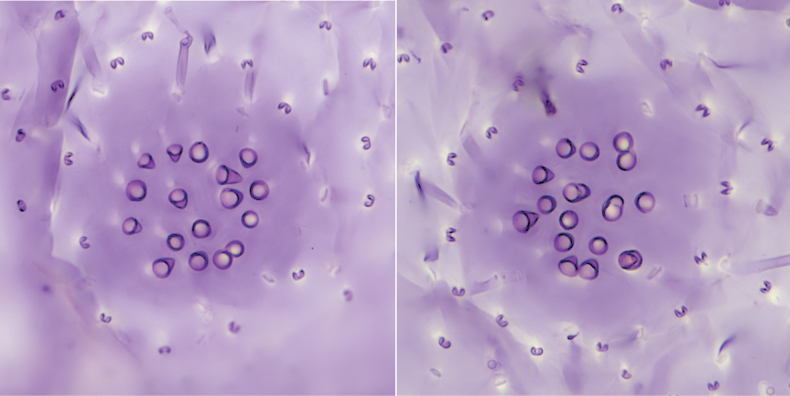
Dorsal cribriform plates of *Lecanodiaspisjiangxiensis* Zhang & Wang, sp. nov.

**Figure 5.**
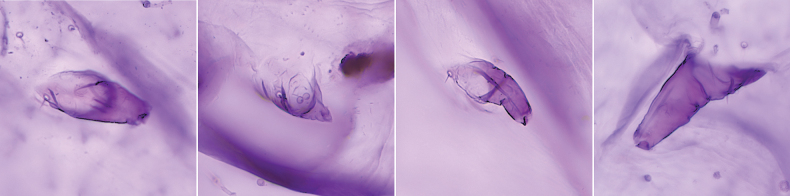
Legs of *Lecanodiaspisjiangxiensis* Zhang & Wang, sp. nov.

Figs 1 , 2 , 3 , 4 , 5

##### Type material.

***Holotype***: • Adult ♀ (mounted singly on a slide, CFJAU), CHINA: Jiangxi Province, Yichun City, Fengxin County / Zaoxia Town, Niyang Village / (28°49'16"N, 115°5'40"E) / on branches of *Castaneamollissima* (Fagaceae) /13.vii.2022, coll. Jiangtao Zhang. ***Paratypes***: • 13 ♀♀ (including two specimens split into dorsal and ventral surfaces and one damaged specimen lacking anal ring, mounted singly on 13 slides, CFJAU), same data as holotype; • 2 ♀♀ (mounted singly on two slides, SWFU), same data as holotype.

##### Description.

***Appearance of adult female in life*** (Fig. 1). Rounded to oval, slightly to strongly convex (becoming more convex with age), yellow to brownish-yellow. With a longitudinal medial carina between 8–10 short conical projections, and ~ three longitudinal lateral carinae on each side.

***Male test*.** Not seen.

***Slide*-*mounted adult female*** (Fig. 2) (*n* = 8): Body oval to subcircular, 4.05–4.95 mm long and 3.75–4.70 mm wide.

***Dorsal surface*. 8-*shaped pores*** (Fig. 2X, V): Numerous, each pore bent, distributed throughout derm, of two sizes. Larger pores (Fig. 2X), each 5–6 μm long, present in a marginal band ~ 2–4 wide, also in irregular clusters on dorsum. Smaller pores (Fig. 2V), each 3.5–4.5 μm long, irregularly spaced throughout remainder of derm. Trilocular pores (Fig. 2W) occasionally present.

***Simple disc pores*** (Fig. 2T): Numerous, evenly distributed, each 2–3 μm in diameter.

***Tubular ducts*** (Fig. 2Q): Numerous, evenly distributed, cylindrical, each 27–36 μm long and 3.5–4.0 μm wide.

***Setae***: Marginal setae (Fig. 2S) stiff, spine-like and pointed apically, each with a stout basal socket, 12.5–20.0 μm long. Other short setae rare dorsally, but not shown on Fig. 2.

***Spiracular setae*** (Figs 2U, R, 3A, B): Two anterior spiracular setae (Figs 2U, 3A), subequal in size, 67.5–85.0 μm long and 7.5–10.0 μm wide, both appearing somewhat concave and bladelike at apex. Two posterior spiracular setae present singly, with one at each outer end of bifid posterior spiracular furrow (Figs 2R, 3B), each seta approximately same length as an anterior spiracular seta.

***Cribriform plates***: Arranged in two longitudinal rows with four plates in each row. Each plate (Figs 2P, 4) 67.5–75.0 μm in diameter and bearing 15–32 small conical-like setae, sometimes with two or three small conical setae fused together.

***Anal ring*** (Fig. 2O): Elliptical, slightly open at posterior end, 60–75 μm in diameter; bearing 10 long setae (12 setae on one specimen), each seta 142–187 μm long, and with three concentric rows of nipple-like projections.

***Anal plates*** (Fig. 2O): Narrowly connected at mid-line. Each plate triangular, 150–172 μm long and 125–245 μm wide, wrinkled dorsally and with 3–5 pores; also bearing two fairly stout setae (occasionally with two on one side and three on the other) situated dorsolaterally on posterior half of plate, each seta 20–25 μm long; and with one slender seta on inner margin, 17.5–32.5 μm long, and two short, slender setae at junction of anal lobes, 17.5–20.0 μm long.

***Arched plate*** (Fig. 2O): Crescent-shaped, 125–150 μm long.

***Anal cleft***: Distinct, 175–300 μm long.

***Anal lobes***: Each lobe slightly rounded, terminating with an approximately apical seta, 75–100 μm long, and 9–14 shorter associated setae, each 15.0–37.5 μm long.

***Ventral surface*. *Antennae*** (Fig. 2A): Well-developed, each usually 9-segmented (1 specimen with segment IV on one side apparently divided into 2 segments; and another specimen with only 1 complete antenna remaining, reduced to only 5 segments); 365–432 μm long; segment lengths (in μm): I, 42.5–56.5; II, 57.5–75.0; III, 57.5–65.0; IV, 50–65; V, 35.0–52.5; VI, 37.5–47.5; VII, 32.5–41.5; VIII, 22.5–27.5; IX, 13.5–22.5. Setae: segment I without or with one hairlike seta; segment II with one long hairlike seta, one shorter hairlike seta and a sensory pore; segments III, IV, and V each without setae; segment VI usually with one hairlike seta; segments VII and VIII each with a long fleshy seta; and terminal segment (IX) with three long fleshy setae and three or four short slender setae.

***Clypeolabral shield***: 202–220 μm long and 167–200 μm wide.

***Labium*** (Fig. 2D): Triangular, 82.5–95.0 μm long and 95.0–122.5 μm wide, with five short setae on each side.

***Legs*** (Figs 2C, 5): Reduced, fused and irregularly shaped (Fig. 5), 2–4-segmented, each leg 37.5–80.0 μm long; each leg usually with one short seta present at base, sometimes leg apices with claw remnants, occasionally tarsal digitules present (found only on 1 hind leg).

***Spiracles*** (Fig. 2B): Anterior spiracles each 82.5–100.0 μm long and 70.0–92.5 μm wide; posterior pair approximately same size. Each anterior spiracle with 3–14 quinquelocular pores in heavily sclerotized area above peritreme; each posterior spiracle with 5–12 quinquelocular pores in similar position. Spiracular furrows containing primarily quinquelocular pores (Fig. 2E), each 3.5–4.5 μm in diameter, and a few 4-, 6- or 7-locular pores (Fig. 2E) spaced throughout furrows.

**8-*shaped pores*** (Fig. 2K, L): Two sizes of 8 shaped pores present on venter. Larger pores (Fig. 2K), each 5.5–6.5 μm long, bent, present in a narrow marginal band ~ 1–3 pores wide. Smaller, flat 8-shaped pores (Fig. 2L), each 4.5–5.0 μm long, forming a narrow submarginal band three or four pores wide just inside marginal pore band. Trilocular pores (Fig. 2M) occasionally present in this band.

***Bilocular pores*** (Fig. 2H): Each 2.0–2.5 μm long, present in submarginal band, reaching posteriorly almost as far as anal lobes.

***Simple disc pores*** (Fig. 2G): Each 2–3 μm in diameter, less numerous than those dorsally.

***Multilocular disc pores*** (Fig. 2N): Primarily each with ten loculi (occasionally 8, 9, 11 loculi), each 6.5–8.0 μm in diameter, pores usually arranged in six transverse segmental bands across abdomen and one transverse band across cephalothorax; sometimes a few pores present also near mesothoracic leg and on prothorax.

***Tubular ducts***: Slightly narrower than those on dorsum (Fig. 2F), each 32.5–41.0 μm long and 3.0–3.5 μm wide, mainly present on margin. Smaller ducts present posterior to vulvar opening.

***Setae***: With 17–29 slender setae just anterior to vulvar opening, mostly each 7.5–17.5 μm long, but with one pair much longer, each 125–142 μm long. A pair of slender submedian setae (Fig. 2I), each 32.5–75.0 μm long, present on most abdominal segments, also extending to the head. Often with a more-or-less well-defined row of short, stout submarginal setae (Fig. 2J), each 10.0–12.5 μm long, associated with submarginal band of flat 8-shaped pores.

***Microspines***: Present on inner margins of anal lobes; smaller microspines numerous mid-ventrally on posterior half of abdomen, microspines not shown on Fig. 2.

##### Remarks.

*Lecanodiaspisjiangxiensis* sp. nov. is similar to *L.pasaniae* (Borchsenius, 1960) in having the posterior spiracular furrow branched and in feeding on Fagaceae. However, *L.jiangxiensis* differs from *L.pasaniae* (character states for the latter given in parentheses) by having: (i) cribriform plates present in two longitudinal rows (absent); (ii) legs present, three pairs (absent, or only the prothoracic legs present); (iii) terminal antennal segment with three fleshy and three or four slender setae (with ~ three fleshy and six slender setae).

##### Etymology.

The species epithet is formed from the name of the type locality, Jiangxi, combined with the Latin suffix “-*ensis*”, meaning “from”.

### ﻿Key to species of the family Lecanodiaspididae known from China

Adapted and modified from Lambdin et al. 1973; Lambdin and Kosztarab 1973; Tang and Hao 1995.

**Table:** 

1	Anal cleft at least twice as long as anal plate; body margin with prominent spiracular indentations	**2**
–	Anal cleft less than twice length of anal plate; body margin without prominent spiracular indentations	**5**
2	Spiracular setae absent; multilocular disc pores limited to posterolateral clusters by vulva	***Anomalococcuscrematogastri* Green**
–	Spiracular setae present; multilocular disc pores forming transverse rows across abdomen, usually also on thorax	**3**
3	Dorsal cribriform plate groups extending into cephalothoracic region	***Psoraleococcusmultipori* (Morrison)**
–	Dorsal cribriform plate groups not extending into cephalothoracic region	**4**
4	Lateral margin of each anal plate with 3 setae; labium with 8 setae	***Psoraleococcusverrucosus* Borchsenius**
–	Lateral margin of each anal plate with 2 setae; labium with 10 setae	***Psoraleococcuslombokanus* Lambdin & Kosztarab**
5	Spiracular setae absent; each spiracle with ornate filiform sclerotizations, but without sclerosis overhanging peritreme	***Cosmococcuserythrinae* Borchsenius**
–	Spiracular setae present; each spiracle without filiform sclerotizations, but sometimes with sclerosis overhanging peritreme	**6**
6	Anal plates distinctly reticulate on outer margins	**7**
–	Anal plates not reticulate, but often with sclerotized ridges	**16**
7	Anal plates separate (not connected at mid-line), each plate with a notch on inner margin	***Lecanodiaspiscremastogastri* Takahashi**
–	Anal plates connected at mid-line, each plate without a notch on inner margin	**8**
8	Anterior spiracular setae dissimilar, 1 fleshy and 1 spine-like	***Lecanodiaspisfoochowensis* Takahashi**
–	Both anterior spiracular setae fleshy	**9**
9	Body twice as long as wide, tapering at both ends	***Lecanodiaspiselongata* Ferris**
–	Body less than twice as long as wide, elliptical	**10**
10	Anal ring with 10 setae	**11**
–	Anal ring with 8 setae	**13**
11	Ventral bilocular pores distributed throughout	***Lecanodiaspispeni* (Borchsenius)**
–	Ventral bilocular pores restricted to submarginal and submedial areas	**12**
12	Posterior spiracular seta longer than the smallest anterior spiracular seta	***Lecanodiaspiscostata* (Borchsenius)**
–	Posterior spiracular seta not longer than the smallest anterior spiracular seta	***Lecanodiaspissimaoensis* Sun & Shi**
13	Cribriform plates in 2 longitudinal rows	**14**
–	Cribriform plates in 4 longitudinal rows	**15**
14	Dorsum with 3 sizes of 8-shaped pores; terminal antennal segment with 7 setae	***Lecanodiaspiscastanea* Sun & Zhang**
–	Dorsum with 2 sizes of 8-shaped pores; terminal antennal segment with 4 or 5 setae	***Lecanodiaspistingtunensis* (Borchsenius)**
15	Terminal antennal segment with 1 spine-like and 3 fleshy setae, the largest fleshy seta usually curved and with numerous forks	***Lecanodiaspisquercus* Cockerell**
–	Terminal antennal segment with 3 slender and 4 fleshy setae, fleshy setae without forks	***Lecanodiaspislithocarpi* Sun & Shi**
16	Posterior spiracular furrow not divided	**17**
–	Posterior spiracular furrow divided	**18**
17	Anterior spiracular setae unequal in size	***Lecanodiaspisrobiniae* (Borchsenius**)
–	Anterior spiracular setae equal in size	***Lecanodiaspiscircularis* (Borchsenius)**
18	Cribriform plates absent; legs absent, or only the prothoracic legs present	***Lecanodiaspispasaniae* (Borchsenius)**
–	Cribriform plates present, in 2 longitudinal rows; legs present, 3 pairs	***Lecanodiaspisjiangxiensis* Zhang & Wang, sp. nov.**

### ﻿Checklist of Lecanodiaspididae from China

#### ﻿Family Lecanodiaspididae Targioni Tozzetti, 1869

**Type genus.***Lecanodiaspis* Targioni Tozzetti, 1869.

#### ﻿Genus *Anomalococcus* Green, 1902

*Anomalococcus* Green, 1902: 260. Type species: *Anomalococcuscrematogastri* Green, 1902 by monotypy and original designation.

#### ﻿*Anomalococcuscrematogastri* Green, 1902

*Anomalococcuscrematogastri* Green, 1902: 261. Type data: Sri Lanka: Paradeniya, on *Ficusreligiosa*. Syntypes, unknown, Type depository: London: The Natural History Museum, UK.

**Host plant in China.** Unidentified host (Lambdin and Kosztarab 1973).

**Distribution in China.** Taiwan (Kuraru [= Guizaijiao]) (Lambdin and Kosztarab 1973).

#### ﻿Genus *Cosmococcus* Borchsenius, 1959

*Cosmococcus* Borchsenius, 1959: 842. Type species: *Cosmococcuserythrinae* Borchsenius, 1959 by original designation.

#### ﻿*Cosmococcuserythrinae* Borchsenius, 1959

*Cosmococcuserythrinae* Borchsenius, 1959: 845. Type data: China: Yunnan Province, Mangshi, on branches of *Erythrinaindica*. Holotype, unknown, Type depository: St. Petersburg: Zoological Museum, Academy of Science, Russia.

*Cosmococcuseuphorbiae* Borchsenius, 1959: 845. Type data: China: Yunnan Province, near Binchwan [= Binchuan], on the stems of *Euphorbiasplendens*. Synonymized by Wang et al. 2024: 339.

*Cosmococcusalbizziae* Borchsenius, 1960: 244. Type data: China: Yunnan Province, on branches of *Albiziamollis*. Synonymized by Wang et al. 2024: 333.

**Material examined.** China: for details, see Wang et al. (2024).

**Host plants in China.**Apocynaceae: *Cascabelathevetia* (Wang et al. 2024); Euphorbiaceae: *Euphorbiamilii* (Borchsenius 1959), *E.royleana* (Wang et al. 2024); Fabaceae: *Albiziamollis* (Borchsenius 1960), *Bauhiniapurpurea*, *Dalbergiaassamica*, *Erythrinacrista-galli* (Wang et al. 2024), *Er.variegata* (Borchsenius 1959); Malvaceae: *Hibiscusrosa-sinensis* (Wang et al. 2024); Moraceae: *Ficus* sp., *F.altissima* (Wang et al. 2024), *F.benghalensis* (Lambdin and Kosztarab 1973); Myricaceae: *Myricarubra* (Wang et al. 2024); Rosaceae: *Prunus* sp. (Wang et al. 2024); Sapindaceae: *Koelreuteriapaniculata* (Wang et al. 2024).

**Distribution in China.** Yunnan (Baoshan, Dali, Dehong, Honghe, Kunming, Puer) (Wang et al. 2024).

#### ﻿Genus *Lecanodiaspis* Targioni Tozzetti, 1869

*Lecanodiaspis* Targioni Tozzetti, 1869: 261. Type species: *Lecanodiaspissardoa* Targioni Tozzetti, 1869 by monotypy.

*Birchippia* Green, 1900: 450–451. Type species: *Birchippiaanomala* Green, 1900. Synonymized by Green 1901: 295.

*Prosopophora* Douglas, 1892: 207. Type species *Prosopophoradendrobii* Douglas, 1892. Synonymized by Cockerell 1896: 50.

#### ﻿*Lecanodiaspiscastanea* Sun & Zhang, 1991

*Lecanodiaspiscastanea* Sun & Zhang, 1991: 342–343. Type data: China: Yunnan Province, Simao, on *Castaneamollissima*. Holotype, female, Type depository: Shandong: Insect Collections of the Department of Plant Protection, Shandong Agricultural University, China.

**Host plant in China.**Fagaceae: *Castaneamollissima* (Shi et al. 1991).

**Distribution in China.** Yunnan (Simao) (Shi et al. 1991).

#### ﻿*Lecanodiaspiscircularis* (Borchsenius, 1960)

*Prosopophoracircularis* Borchsenius, 1960: 226. Type data: China: Yunnan Province, 25 km north of Tsindun [= Jingdong], on unknown host. Holotype, female, Type depository: St. Petersburg: Zoological Museum, Academy of Science, Russia.

*Lecanodiaspiscircularis* (Borchsenius, 1960): Howell and Kosztarab 1972: 159.

*Prosopophoracircularis* Borchsenius, 1960: Kozár and Drozdják 1998: 418.

**Host plant in China.**Fabaceae: *Trifolium* sp. (Tang and Hao 1995).

**Distribution in China.** Yunnan (Jingdong) (Borchsenius 1960).

#### ﻿*Lecanodiaspiscostata* (Borchsenius, 1959)

*Psoraleococcuscostatus* Borchsenius, 1959: 842. Type data: China: Yunnan Province, 25 km south of Szemao [= Simao], in forest, on the stems of an undetermined plant. Holotype, female, Type depository: St. Petersburg: Zoological Museum, Academy of Science, Russia.

*Lecanodiaspiscostata* (Borchsenius, 1959): Lambdin et al. 1973: 6.

*Psoraleococcuscostatus* Borchsenius, 1959: Kozár and Drozdják 1998: 419.

**Host plant in China.**Fagaceae: *Castaneamollissima* (Lambdin et al. 1973).

**Distribution in China.** Yunnan (Simao) (Borchsenius 1959).

#### ﻿*Lecanodiaspiscremastogastri* Takahashi, 1929

*Lecaniodiaspis* [sic!] *cremastogastri* Takahashi, 1929: 47. Type data: China: Taiwan Province, Hori [= Howli], attacking branches of *Lithocarpus*. Syntypes, female, Type depository: Sapporo: Entomological Institute, Faculty of Agriculture, Hokkaido University, Japan.

*Lecanodiaspiscremastogastri* Takahashi, 1929: Howell and Kosztarab 1972: 122.

**Host plants in China.**Fagaceae: *Lithocarpus* sp. (Takahashi 1929), *Quercus* sp. (Takahashi 1942).

**Distribution in China.** Hainan (Dwa Bi) (Takahashi 1942), Taiwan (Howli) (Howell 1971).

#### ﻿*Lecanodiaspiselongata* Ferris, 1950

*Lecaniodiaspis* [sic!] *elongata* Ferris, 1950: 6. Type data: China: Yunnan Province, near Kunming, An-lin-wen-chian [= Anning], on *Lithocarpusspicata*. Syntypes, female, Type depository: Davis: The Bohart Museum of Entomology, University of California, California, USA.

*Lecanodiaspiselongata* Ferris, 1950: Howell and Kosztarab 1972: 127.

**Material examined.** • 4 ♀♀, China: Yunnan Province, Chuxiong Yi Autonomous Prefecture, Zixishan Forest Park, Baotouwang (25°2'33"N, 101°26'21"E), on the back of *Quercuslongispica* (Fagaceae) leaves, 14.vii.2021, coll. Hongfei Zhang, Zeren Tan.

**Host plants in China.**Fagaceae: *Lithocarpuselegans* (= *L.spicatus*) (Ferris 1950), *Quercuslongispica*.

**Distribution in China.** Yunnan (Chuxiong, Kunming (Ferris 1950)).

#### ﻿*Lecanodiaspisfoochowensis* Takahashi, 1936

*Lecaniodiaspis* [sic!] *cremastogastrifoochowensis* Takahashi, 1936: 217. Type data: China: Fujian Province, Foochow [= Fuzhou], on an unknown host. Lectotype, female, by subsequent designation. Type depository: Sapporo: Entomological Institute, Faculty of Agriculture, Hokkaido University, Japan.

*Lecanodiaspisfoochowensis* Takahashi, 1936: Howell and Kosztarab 1972: 127.

*Psoraleococcusfoochowensis* (Takahashi, 1936): Kozár and Drozdják 1998: 420.

**Host plant in China.** Unidentified host (Lambdin et al. 1973).

**Distribution in China.** Fujian (Fuzhou) (Takahashi 1936).

#### ﻿*Lecanodiaspisjiangxiensis* Zhang & Wang, sp. nov.

**Material examined.** • 16 ♀♀, China: Jiangxi Province, Yichun City, Fengxin County, Zaoxia Town, Niyang Village (28°49'16"N, 115°5'40"E), on branches of *Castaneamollissima* (Fagaceae), 13.vii.2022, coll. Jiangtao Zhang. Holotype, female. Type depository: Jiangxi: College of Forestry, Jiangxi Agricultural University, China.

**Host plant in China.**Fagaceae: *Castaneamollissima*.

**Distribution in China.** Jiangxi (Yichun).

#### ﻿*Lecanodiaspislithocarpi* Sun & Shi, 1991

*Lecanodiaspislithocarpi* Sun & Shi, 1991: 185–188. Type data: China: Yunnan Province, Simao, on *Lithocarpuscorneus*. Holotype, female, Type depository: Shandong: Insect Collections of the Department of Plant Protection, Shandong Agricultural University, China.

**Host plant in China.**Fagaceae: *Lithocarpuscorneus* (Sun and Shi 1991).

**Distribution in China.** Yunnan (Simao) (Sun and Shi 1991).

#### ﻿*Lecanodiaspispasaniae* (Borchsenius, 1960)

*Prosopophorapasaniae* Borchsenius, 1960: 224. Type data: China: Yunnan Province, on branches of *Pasaniadealbata*. Holotype, female, Type depository: St. Petersburg: Zoological Museum, Academy of Science, Russia.

*Lecanodiaspispasaniae* (Borchsenius, 1960): Howell et al. 1973: 46.

*Prosopophorapasaniae* Borchsenius, 1960: Kozár and Drozdják 1998: 418.

**Host plant in China.**Fagaceae: *Lithocarpusdealbatus* (Borchsenius 1960).

**Distribution in China.** Yunnan (Kunming) (Borchsenius 1960).

#### ﻿*Lecanodiaspispeni* (Borchsenius, 1960)

*Prosopophorapeni* Borchsenius, 1960: 229. Type data: China: Sichuan Province, on branches of unknown tree. Holotype, female, Type depository: St. Petersburg: Zoological Museum, Academy of Science, Russia.

*Lecanodiaspispeni* (Borchsenius, 1960): Lambdin et al. 1973: 20.

*Prosopophorapeni* Borchsenius, 1960: Kozár and Drozdják 1998: 419.

**Host plant in China.** Unidentified host (Borchsenius 1960).

**Distribution in China.** Sichuan (Chengdu) (Borchsenius 1960).

#### ﻿*Lecanodiaspisquercus* Cockerell, 1896

*Lecaniodiaspis* [sic!] (*Prosopophora*) *quercus* Cockerell, 1896: 51. Type data: Japan: Tokyo, on *Quercus*. Syntypes, female, Type depository: Washington: United States National Entomological Collection, U.S. National Museum of Natural History, District of Columbia, USA.

*Prosopophoraquercus* (Cockerell, 1896): Maskell 1897: 243.

*Psoraleococcusquercus* (Cockerell, 1896): Borchsenius 1960: 241.

*Lecanodiaspisquercus* Cockerell, 1896: Howell and Kosztarab 1972: 127.

*Lecanodiaspismajesticus* Wang & Qiu, 1986: 303. Type data: China: Shandong Province, on *Pasaniacleistocarpa*. Synonymized by Tang and Hao 1995: 268.

**Material examined.** • ♀, China: Yunnan Province, Zhaotong City, Daguan County, Tianxing Town, Nanmakan (27°50'17"N, 103°58'12"E), on the branch of *Quercusacutissima* (Fagaceae), 20.iv.2021, coll. Xubo Wang.

**Host plants in China.**Fagaceae: *Lithocarpuscleistocarpus* (= *Pasaniacleistocarpa*) (Wang and Qiu 1986), *Quercusacutissima*.

**Distribution in China.** Shandong (Wang and Qiu 1986), Yunnan (Zhaotong).

#### ﻿*Lecanodiaspisrobiniae* (Borchsenius, 1960)

*Prosopophorarobiniae* Borchsenius, 1960: 230. Type data: China: Yunnan Province, vicinity of Kunming, on branches of *Robiniapseudoacacia*. Holotype, female, Type depository: St. Petersburg: Zoological Museum, Academy of Science, Russia.

*Lecanodiaspisrobiniae* (Borchsenius, 1960): Howell and Kosztarab 1972: 2.

*Prosopophorarobiniae* (Borchsenius, 1960): Kozár and Drozdják 1998: 419.

**Host plant in China.**Fabaceae: *Robiniapseudoacacia* (Borchsenius 1960).

**Distribution in China.** Yunnan (Kunming) (Borchsenius 1960).

#### ﻿*Lecanodiaspissimaoensis* Sun & Shi, 1991

*Lecanodiaspissimaoensis* Sun & Shi, 1991: 341–342. Type data: China: Yunnan Province, Simao, on *Quercus*. Holotype, female, Type depository: Shandong: Insect Collections of the Department of Plant Protection, Shandong Agricultural University, China.

**Host plant in China.**Fagaceae: *Quercus* sp. (Shi et al. 1991).

**Distribution in China.** Yunnan (Simao) (Shi et al. 1991).

#### ﻿*Lecanodiaspistingtunensis* (Borchsenius, 1960)

*Prosopophoratingtunensis* Borchsenius, 1960: 231. Type data: China: Yunnan Province, vicinity of Tsindun [= Jingdong], on branches of *Pasania*. Holotype, female, Type depository: St. Petersburg: Zoological Museum, Academy of Science, Russia.

*Lecanodiaspistingtunensis* (Borchsenius, 1960): Lambdin et al. 1973: 24.

*Prosopophoratingtunensis* Borchsenius, 1960: Kozár and Drozdják 1998: 419.

**Material examined.** • 14 ♀♀, China: Yunnan Province, Puer City, Ninger Hani and Yi Autonomous County, near People’s Hospital (23°3'39"N, 101°2'26"E), on the branches of *Elaeocarpusdecipiens* (Elaeocarpaceae), 17.x.2018, coll. Xubo Wang.

**Host plants in China.**Elaeocarpaceae: *Elaeocarpusdecipiens*; Fagaceae: *Lithocarpus* sp. (= *Pasania* sp.) (Borchsenius 1960).

**Distribution in China.** Yunnan (Jingdong (Borchsenius 1960), Puer).

#### ﻿Genus *Psoraleococcus* Borchsenius, 1959

*Psoraleococcus* Borchsenius, 1959: 841. Type species: *Psoraleococcusverrucosus* Borchsenius, 1959.

#### *Psoraleococcuslombokanus* Lambdin & Kosztarab, 1973

*Psoraleococcuslombokanus* Lambdin & Kosztarab, 1973: 63. Type data: Indonesia: Lombok, at Rampoeng (45 km east of Mataram), on *Annonamuricata*. Holotype, female, Type depository: Washington: United States National Entomological Collection, U.S. National Museum of Natural History, District of Columbia, USA.

**Host plants in China.**Fagaceae: *Quercus* sp. (Tang and Hao 1995); Myrtaceae: *Eucalyptus* sp. (Tang and Hao 1995).

**Distribution in China.** Fujian (Fuzhou) (Tang and Hao 1995), Sichuan (Mount Emei) (Tang and Hao 1995).

#### ﻿*Psoraleococcusmultipori* (Morrison, 1921)

*Anomalococcusmultipori* Morrison, 1921: 641. Type data: Singapore: on *Nepheliumlappaceum*. Holotype, unknown, Type depository: Washington: United States National Entomological Collection, U.S. National Museum of Natural History, District of Columbia, USA.

*Psoraleococcusmultipori* (Morrison, 1921): Lambdin and Kosztarab 1973: 73.

**Material examined.** • 9 ♀♀, China: Hainan Province, Wuzhishan City, Tongshen Town (18°44'12"N, 109°32'32"E), on the branches of *Dimocarpuslongan* (Sapindaceae), 20.ii.2022, coll. Chaoqun Li, Huihui Zhong, Jiangtao Zhang.

**Host plant in China.**Sapindaceae: *Dimocarpuslongan*.

**Distribution in China.** Hainan (Wuzhishan).

#### ﻿*Psoraleococcusverrucosus* Borchsenius, 1959

*Psoraleococcusverrucosus* Borchsenius, 1959: 842. Type data: China: Yunnan Province, 8 km north of Szemao [= Simao], in forest, on stems of an undetermined stems. Holotype, female, Type depository: St. Petersburg: Zoological Museum, Academy of Science, Russia.

**Material examined.** • 5 ♀♀, China: Yunnan Province, Dai Autonomous Prefecture of Xishuangbanna, Mengla County, Menglun Town, Xishuangbanna Tropical Botanical Garden (21°55'41"N, 101°15'26"E), on the branches of *Calamus* sp. (Arecaceae), 20.iv.2018, coll. Xubo Wang; • 2 ♀♀, China: Hainan Province, Ledong Li Autonomous County, Jianfengling Rainforest Valley (18°44'45"N, 108°55'57"E), on the branches of *Ilexchinensis* (Aquifoliaceae), 15.iii.2021, coll. Yong Wang.

**Host plants in China.**Aquifoliaceae: *Ilexchinensis*; Arecaceae: *Calamus* sp.; Phyllanthaceae: *Glochidion* sp. (Lambdin and Kosztarab 1973).

**Distribution in China.** Hainan (Ledong), Taiwan (Kuraru [= Guizaijiao], Taihoku [= Taipei]) (Lambdin and Kosztarab 1973), Yunnan (Simao (Borchsenius 1959), Xishuangbanna).

## ﻿Discussion

Wang (1982) established the genus *Crescoccus* with *C.candidus* Wang, 1982 as the type species, and placed it within the family Lecanodiaspididae. *Crescoccus* is distinguished from other genera of Lecanodiaspididae by the following characters: (i) absence of stigmatic spines; (ii) anal ring with six setae; (iii) a distinctive type of cribriform plates; (iv) absence of multilocular disc pores; (v) numerous quinquelocular pores over the entire dorsum (Wang 1982). However, Tang and Hao (1995) suggested that *C.candidus* is actually the nymph of *Pseudopulvinariasikkimensis* Atkinson, 1889 (family Coccidae), and treated it as a synonym of the latter. Since no original specimens of *C.candidus* are available for examination, the genus *Crescoccus* and the species *C.candidus* were excluded from this study.

Douglas (1892) proposed the genus *Prosopophora* with *P.dendrobii* Douglas, 1892 as the type species. Later, Cockerell (1896) synonymized it under *Lecanodiaspis*. In 1960, Borchsenius (1960) re-established the genus and added several new species. Subsequently, Howell and Kosztarab (1972) made a thorough revision of *Lecanodiaspis* and once again synonymized *Prosopophora* with *Lecanodiaspis*. However, Kozár and Drozdják (1998) continued to treat *Prosopophora* as a valid genus, but apparently without having studied the original specimens and therefore without a reliable basis for its separation from *Lecanodiaspis*. Since Howell and Kosztarab’s (1972) revision was mainly based on original specimens, their work, at least for now, provides the most reliable distinctions between related species and genera. In this study, we follow the conclusions of Howell and Kosztarab (1972), while acknowledging that future methods, such as molecular data, may provide more clarity. For the benefit of future researchers, we have added the citation of Kozár and Drozdják (1998) in the species nomenclatural histories, although their suggestions were not adopted in this study.

Wang and Qiu (1986) reported a *Lecanodiaspis* species, *L.majestica* Wang & Qiu, 1986, collected on *Lithocarpuscleistocarpus* (= *Pasaniacleistocarpa*) (Fagaceae) from Shandong, China. The species is similar to *L.quercus* Cockerell, 1896, but can be distinguished by the following characters: (i) terminal antennal segment with four spine-like setae, with the largest seta sometimes forked; (ii) cribriform plates arranged in four longitudinal rows, evenly scattered on the meta- and mesothorax; (iii) absence of legs (Wang and Qiu 1986). However, Tang and Hao (1995) found that none of the above characters were beyond the range of variation described by Howell and Kosztarab (1972) with specimens of *L.quercus* mainly from Japan, so they treated it as a junior synonym of the latter. Here, we follow the opinion of Tang and Hao (1995).

*Lecanodiaspiscostata* (Borchsenius, 1959) was originally reported from Simao, China on an undetermined plant by Borchsenius (1959) under the name *Psoraleococcuscostatus*. Later, Tang and Hao (1995) recorded this species from Mengyang, feeding on *Quercusacutissima* and *Q.variabilis* (Fagaceae). However, since they did not provide details of the specimens examined, the distribution and host plants they provided are not included in our checklist.

*Psoraleococcusmultipori* (Morrison, 1921) was first reported by Morrison (1921) from Singapore on *Nepheliumlappaceum* (Sapindaceae) under the name *Anomalococcusmultipori*. The species was later recorded from Indonesia (Green 1930) and Philippines (Lit 1997). Takahashi (1928) had previously recorded this species from the nest of *Cremastogasterrogenhoferi* (family Formicidae) in Taiwan, but this was later found to be an undescribed species (Takahashi 1929). More recently, Miller et al. (2014) examined specimens taken at United States quarantine inspection, including some from Taiwan. However, the origin of these specimens remains uncertain (D.R. Miller and S.A. Schneider pers. comm.). Therefore, the specimens collected in Hainan during the present study represent the first confirmed record of this species in China.

## Supplementary Material

XML Treatment for Lecanodiaspis

XML Treatment for Lecanodiaspis jiangxiensis

## ﻿Additional information

### Author ORCIDs

Jiangtao Zhang https://orcid.org/0000-0002-0007-3918

Keqing Wang https://orcid.org/0009-0001-2308-2948

Gillian W. Watson https://orcid.org/0000-0001-9914-0094

Xingping Liu https://orcid.org/0000-0003-1831-9290

Xubo Wang https://orcid.org/0000-0001-5996-6027

### Data availability

All of the data that support the findings of this study are available in the main text.
